# Proceedings of the American Medical Association

**Published:** 1875-06

**Authors:** 


### Our Exchanges.

---

PROCEEDINGS OF THE AMERICAN MEDICAL ASSOCIATION—*Twenty-sixth Annual Session: Louisville, May* 4-7, 1875.—The meeting was called to order at 11 A.M. on Tuesday, May 4th. Dr. Toner, the former President, presented his successor, Dr. Bowling to the Association. Dr. E. Richardson, the chairman of the Committee of Arrangements, delivered an eloquent address of welcome, dilating freely, but not disagreeably, on the claims of his State to medical distinction, alluding to the eminent writers and practitioners, among whose names are those of Brashear, McDowell, Briggs and Dudley, and concluding by a hearty welcome to the old Kentucky home. He announced that the Association would meet in convention each day from 9:30 A.M. to 1 P.M., and the different sections separately at 3 P.M.

Dr. Davis, of Chicago, announced that Dr. Bottsford, President of the Canadian Medical Association, was present, and moved that he be invited to a seat on the platform. The motion was carried, and, upon complying, Dr. Bottsford addressed the Association as follows:

GENTLEMEN: I have been called upon by your President to address you upon this occasion. The fewer the words I say will no doubt make it the more acceptable. As you are aware, the country which I have the honor to represent extends along your northern border, touching the Atlantic on the east and the Pacific on the west. I have come to learn the principles which have secured your success—a success manifest from the assemblage which I see before me. We are as yet a few in number, scattered over a large surface, and we are making an effort to advance the interest of our profession. These efforts as yet cannot be said to show great results, but we trust before long to enter the arena and do battle, not with the arms of conflict, but in the cause of the principles which are the foundation of our profession, and as benefactors of the race; for, without arrogating to ourselves undue prominence, I think I might, without excepting any body of men or profession, claim for medical men an amount of self-denial and sympathy for their fellow-beings not to be surpassed or even equaled.

Some amusement was caused at the close of Dr. Bottsford's remarks, by a wish from a Texas delegate that Canada might have the good fortune that befell Texas, and be annexed to the United States.

The President then delivered an oration, of which the following is an abstract:

A notional association of medical men was without precedent when this was ushered into existence by the genius of one man, watered by his parental solicitude, and sustained by the co-operation of his brethren, all stimulated by a common hope that good would come of it in cementing the brotherhood in unity of purpose, and intensifying its power for the achievement of good to the profession, and consequently to the public at large. Thus organized, and freighted with the hopes and blessings of every loyal medical heart in the country, it has literally drifted through a generation. Composed of the representatives of wide-spread and independent medical masses, with many-sided hopes and aspirations, many with a freedom of thought and expression peculiar to their latitudes, it has seemed, in turn, to delight in representing every shade of medical politics. But it still lived, and every year its ancient friends met new representatives in council, and, renewing their allegiance, lighted again their torches at its altar. The contributions of the old and new were printed, and, in a bound book, sanctified to posterity.

It is good occasionally to recall the grand objects its founders hoped to achieve through its instrumentality. They were—

1. To give emphatic expression to the views and aims of the medical profession of this country.

2. To supply more effectual means than have hitherto been available here for cultivating and advancing medical knowledge.

3. To elevate the standard of medical education.

4. To promote the usefulness, honor and interest of the medical profession.

5. To enlighten and direct public opinion in regard to the duties, responsibilities and requirements of medical men.

6. To excite and encourage emulation and concert of action in the profession.

7. To facilitate and foster friendly intercourse between medical men.

8. To take cognizance of the common interest of the medical profession in every part of the United States.

After eulogizing briefly the success the Association had met with in the pursuit of the first two objects, the orator enlarged on the third point. The past policy of the Association was sketched as follows:

At Nashville, eighteen years ago, amid a storm of school representatives in this Association, a resolution was introduced to so change our constitution as to keep the representatives of schools and hospitals, as such, out of this body. Under the rule, it must wait a year for consideration. It was called up the next year at Washington, after a great excitement about hospital representatives, and was lost by an almost unanimous vote. In 1869, at New Orleans, the same proposition was made. A greater storm at the meeting in Washington, in 1870, from school representatives, caused deeper thought upon the subject, and at Detroit, last year, seventeen years after the Nashville resolution, to the unspeakable joy of many, the constitution was so amended as to give a permanent quietus to this disturbing element, and assurance of a calmer future.

Other changes of less importance have, from time to time, lent their aid toward securing for the society as much of finish and beauty as are compatible with the imperfection of the human understanding. By the aid of committees, all disturbing influences, such as once convulsed the assembly, are quietly disposed of, and a stranger present during hours of business would regard it as equal in dignity and decorum to any representative body in the world. Though a little late, perhaps, in arriving at the full proportion and stature of manhood, the induration of its ligaments, fusion and condensation of parts, with general unity and individuality, are doubtless the more perfect, and, in consequence, give earnest of prolonged youth and an old age that shall know no decay.

If this body has not of itself accomplished all its friends hoped for in the beginning, in elevating the standard of medical education, they must be satisfied to know that that standard, notwithstanding, has been regularly going up, fully abreast with the progress of our new country in every other department of human learning, and all the arts and appliances of a rapidly developing civilization. The spring can only well up the waters sent to it, purifying them in the process, and the sea is but the representative of many waters. The schools must take such material as they can get, and make the most of it; and the American Medical Association, as in the past, so now and hereafter, is obliged to consist of such representative medical men as the schools may prepare and fashion for its use. The stream cannot rise higher than its sources.

That the schools are all that their hopeful, faithful and earnest teachers can make them, and that they accomplish all that is possible with the material intrusted to them, none ought to doubt; and that the country at large selects as good material as it possesses for the schools is equally indisputable. Nor should any believe that the youth selected for medical schools are, in respect of preparatory education, a whit inferior to those selected for the law or divinity.

The question returns to us, What can the Association now do, in its early manhood, honestly, toward redeeming implied pledges in its infancy? Much, if it have nerve or backbone; nothing, if these be absent. The plan is simple, as all plans are that succeed. Let it be solemnly resolved by this meeting that it shall be regarded as derogatory to the character of any physician, in any part of the United States, to take under his care as a student of medicine any one who cannot exhibit evidence of having taken a degree in a regularly chartered college or a certificate of qualifications necessary to become a student of medicine, from a board of examiners appointed for that purpose by the American Medical Association. This will do the work.

Territories and new States, in a country like ours, in a formative state, will provide themselves with medical helps in the mode we have described, which, existing outside of this body, and independent of it, will occasion it no concern whatever. Nor would the schools suffer pecuniary loss under this rule. When it was generally known, as it soon would be, young men desiring to enter the profession would earnestly devote themselve to the duties of preparation, nor relax their efforts till possessed of the degree or the certificate.

Let the doctorate imply something more than "two full courses of lectures, the last of which must be in this institution." Besides, it would give the college an ample excuse for not receiving every uneducated, lazy dolt who desired to make a living under false pretenses.

Neither would this rule exclude any one from being a doctor. In a vigorous republic there will always spring up men who, by genius and long self-training, literally hew their way to greatness, in all of the professions, while many more will pass through colleges, winning all their honors, to sink into insignificance, and to go through the world unknowing and unknown. For the former, Heaven has made ample provision, and stamped them as the nobility of nature, whom this body can neither depress nor elevate; nay, nor could an association of angels.

The speaker, after alluding to the progress of the profession in late years, closed with a characteristically fervent tribute to some of the prominent members of the Association.

The Secretary read a note from the Vice-President, Dr. H. W. Brown, of Texas, apologizing for his absence.

The meeting then adjourned.

In the afternoon Dr. Nathan Allen, of Lowell, read a paper before the section of Obstetrics, in which he claimed that the truly normal standard of womanhood was that of the highest anatomical development and of the greatest physiological excellence. The essay elicited considerable criticism.

The ball and banquet at the Galt House, in the evening, was a perfect success, which we regret we have no space to dwell upon. The medical editors were entertained at dinner by Dr. D. W. Yandell.

WEDNESDAY'S PROCEEDINGS.

Wednesday, May 5th, the Secretary read the names of newly arrived delegates, which swelled the total to four hundred and thirty-five. The delegates of each State then appointed one of their number to form part of the nominating committee. Extracts were read from the records of the Canadian Medical Association, declaring it the unanimous opinion of that body that an internatianal convention of the associations of America and Canada would be beneficial to both.

Dr. Toner, chairman of the committee to whom the matter was referred at the last meeting, presented a report including the following resolutions, which were adopted:

*Resolved,* That the Association learn with regret that no action was taken by the last Congress upon its recommendation in behalf of the medical department of the United States army, and that we respectfully renew our petition that Congress will enact such a bill for the benefit of the medical department of the army, as well as secure to its officers that share of rank and promotion to which we consider that they are entitled, and which should be at least fully equal to that enjoyed by any other staff corps, or by the medical corps of the navy.

*Resolved,* That a committee of five be appointed to call the attention of Congress to this subject, and to the petitions which were forwarded to the last Congress by the physicians of the United States.

Dr. Seguin, of New York, read the following paper:

*To the American Medical Association:*

Mr. President and Gentlemen: You have twice sent delegates to the British Medical Association, and kindred European societies, to invite them to concert a plan of uniformity of methods, instruments, scales, and records for clinical observation.

This proposition has become more opportune since the meeting, in Paris, of the convention for the adoption of uniform weights and measures by all nations, in which convention Profs. Henry and Hilgord represent the United States, but in which the special wants of unity of measures of our profession are not requested.

It was advocated by Sir William Jenner, MM. Reynolds, Gibson, Stewart, Squire, Sydney Ringer, Wilson, and Tilbury Fox, in England; on the Continent, by MM. Morey, Charcot, Lorain, Potain, Lepine, Ollier, all ready to open a commission in Paris and a sub-committee in Lyons, in order to concur in your plan of uniform observation.

This plan embraces the unity of clinical thermometers and of thermometric scales, charts, etc., a uniform graduation of the sphygmograph, myograph, spirograph, æsthesiometer, manometer, globulimeter, ophthalmoscope, thermoscope, and other instruments of precision used in diagnoses, a uniform method of measuring and registering the hearing, the velocity of other sensory impressions, the regularity of co-ordinate movements, as the walk, and a uniform registration of all clinical cases according to their kind.

Of this plan the International Medical Congress, meeting at Brussels the 19th September prox., proposes to carry out only one part: the uniform measurement and record of hearing by all nations. It is therefore important that the American Medical Association be represented this year at Brussels, in order to represent there the original plan of uniformization of clinical observation in its integrity and entirety.

Therefore, the American Medical Association resolves to nominate new delegates, commissioned to again advocate in Europe the unity of clinical observation, and charge them to report progress in brief, at the next meeting of 1876.

One of the delegates, E. Seguin.

The resolution was adopted.

Dr. Gross, in accordance with the permission voted on the preceding day, then delivered an address on Blood-Letting, which he termed a lost art. After stating the sway that this practice had held for two thousand years, and dwelling on its popularity and universal application, he spoke of the almost total disuse into which it has fallen, and remarked that it behooves us to inquire whether there is not something wrong in the discontinuance of the practice, and whether we have not fallen into the opposite error. Extremes are always dangerous, and they are especially dangerous in the practice of medicine. It behooves us to be on the watch. If the new way is right, the old was certainly wrong. The lancet is now an obsolete instrument. The office of cupping has departed, and blood-letting is emphatically a lost art.

He then proceeded to consider the causes of the loss, which were four, namely:

1. The influence or tyranny of fashion.
2. The indiscriminate use of the lancet.
3. The acquirement of a more accurate knowledge of disease.
4. Knowledge of medicines hitherto unknown.

After discussing these causes in detail, Dr. Gross spoke strongly in favor of bleeding for many diseases, especially for inflammatory ones; the bleeding, however, to be performed early. He predicted that bleeding would again come to be recognized as a therapeutic agent, but that it would not be practiced indiscriminately.

At the conclusion of his remarks he was loudly applauded.

Then followed the address of the president of the section on Materia Medica and Physiology, which was well delivered and received.

The report of the committee on the International Medical Association was read and referred to the committee on nominations. The report recommended the establishment of such an association in this country, but at the same time thought it advisable to defer the matter for a time. Sending delegates to the convention to assemble at Brussels was thought the most expedient plan to meet the wants felt by the members of the American Association.

The committee appointed to select a die, with the portrait of Dr. N. S. Davis on one side and the name and date of the Association on the other, reported that they had arranged for the manufacture of the same in bronze, at the Philadelphia mint. The report was received, and the committee instructed to order two hundred medals at $1 each; twelve cents extra for postage.

The address of the president of the section on Practical Medicine, Dr. Austin Flint, of New York, was then announced amid the plaudits of the assembly. He began by saying that he had the honor to submit a rather imperfect report upon medical discoveries for the past year.

The subject-matter of the essay referred to alcoholism, motor centers, new remedial agents, transfusion of blood, and the natural history of crime. The changes of alcohol in the system and its medicinal uses were dwelt upon at some length. Some held that alcohol passes into the blood and is expelled through the emunctories unchanged; while others denied this, and held that it was appropriated by the animal economy. Well-conducted experiments, however, went to prove that when alcohol was thus taken into the system, the proportion excreted by the kidneys, lungs and skin is exceedingly small, the greater part being destroyed in the body. What becomes of it? This remains to be answered by further experimental researches. Six hundred grains of absolute alcohol can be disposed of without injury to the bodily functions of a healthy adult. It is accordingly employed in the treatment of many conditions of disease, though its use is not based upon any ascertained facts concerning its elimination.

The physiological investigations during the past year in relation to motor centers of the brain-convulutions were then touched upon, and likewise with reference to newly-discovered remedial agents, when the lecturer passed on to the consideration of the transfusion of blood.

While there were many experiments performed in the transfusion of the blood of one animal into the veins of another of unlike genus, and of the blood of a lamb into the veins of a man, himself a physician in one instance, there were certain curious results noticed, but nothing positive had been elaborated that would justify the positive advocacy of any reliable feature or theory of practice. The subject was not without interest or promise, however, and afforded an ample field for any one whose zeal for the advancement of medical knowledge in that direction was equal to the task of an investigation, and which could hardly fail, eventually, to be of signal advantage to the profession.

The closing feature of the paper had reference to the natural history of crime, in which a query was announced concerning the possible connection of individual tendencies to the commission of crime with corresponding diseased conditions of the organization.

The bulk of the essay embraced the consideration of this last topic.

The paper was referred to the section of Practical Medicine, and will be published in the annual report of the Association.

By a unanimous vote, Dr. Flint was authorized to continue the researches alluded to in his paper.

The convention then adjourned till ten o'clock on Thursday morning.

THURSDAY'S PROCEEDINGS.

The meeting was called to order at ten o'clock, by the President, Dr. Bowling. Delegates were appointed to represent the American Association in the International Medical Association to be held in Brussels, in September, 1875, and to confer with a committee from the Canadian Medical Association, which will meet in Halifax, August 5, 1875, on the subject of holding an international convention by the two associations.

The report of the committee on publication was received.

The Treasurer's report stated that it would be necessary to be very guarded as to the amount of matter admitted into the journal of the proceedings, or it would be found impossible to embrace it all in the report without increasing the price of preparing it. Owing to the advance recently made in the rate of postage, the reports would be delivered at six dollars instead of five dollars, the price heretofore charged. Three thousand and twenty-two dollars and forty-one cents is the amount reported in the treasury at this time. This report, as was also the Librarian's, was received.

Dr. J. Marion Sims rose from his seat upon the disposal of these reports, and stated that he desired to offer the report of the committee on the McDowell Memorial Fund. He then briefly traced the history of the movement, concluding with the following resolutions:

WHEREAS, It is universally acknowledged that the late Ephraim McDowell, of Kentucky, was the originator of ovariotomy; and

WHEREAS, We believe that proper measures should be instituted to commemorate this great achievement, and do appropriate honor to its author; therefore,

*Resolved*, That this Association recommend to each of its members, and to the profession generally, to contribute annually such sums as they may think proper, until the amount of ten thousand dollars shall be accumulated, which shall be known as the McDowell Memorial Fund, the interest of which shall be devoted to the payment of prizes for the best essays relating to the diseases of the ovaries.

*Resolved*, That this fund shall be invested by trustees, to be appointed by the Association, and subject to such resolutions as it may desire.

*Resolved*, That this Association shall elect a board of three trustees, whose duty it shall be to carry out the object of these resolutions, and whose term of office shall continue five years.

*Resolved*, That this Association will leave to the State of Kentucky the grateful privilege of providing a local memorial to the memory of Dr. McDowell.

At the conclusion of the reading of the report, Dr. Gross rose and said that he felt desirous of saying a few words in favor of this fund, as it had been his privilege and good fortune to first set forth the claims on this continent of Dr. McDowell to the title of "Father of Ovariotomy."

The resolutions were adopted.

Dr. E. M. Moore, of Rochester, New York, read a paper on Transfusion, in which he favored the direct method of performing the operation. The essay was a learned contribution to the subject.

The Judicial Committee reported upon various matters of ethics which had been referred to them.

Dr. Byford, of Chicago, read an address on Obstetrics.

The Association then adjourned until the next day.

FRIDAY'S PROCEEDINGS.

It was voted to hold the next meeting in Philadelphia, the the first Tuesday in June, 1876.

The following officers for the ensuing year were elected: President, J. Marion Sims, of New York; Vice-Presidents, Dr. John D. Jackson, of Kentucky; Dr. Samuel Lilley, of New Jersey; Dr. N. Pinckney, U. S. A.; Dr. S. D. Seeley, of Alabama; Treasurer, Dr. Caspar Wistar, of Pennsylvania; Librarian, Dr. William Lee, of the District of Columbia; Committee on Library, Dr. Johnson Elliott, of the District of Columbia; Assistant Secretary, Dr. Richard J. Dunglison, of Pennsylvania.

The chairmen and secretaries of the several sections were duly appointed, and the following gentlemen were put on the Judicial Committee, in the place of those whose terms had expired: Dr. L. S. Joynes, Virginia; R. N. Todd, Indiana; Robert Battey, Georgia; James E. Morgan, District of Columbia; Thos. B. Flaylor, New Jersey; Silas N. Benham, Pennsylvania: A. Dunlap, Ohio. The rest of the present council continued.

The following were appointed a committee on prize essays: Drs. Samuel D. Gross, F. G. Smith, Alfred Stille, Ellerslie Wallace, and H. C. Wood, of Pennsylvania. The present committee made their report, which was received without reading, owing to its length, and was referred to a committee of experts, consisting of Drs. Ashurst, Gross and Agnew.

After appropriate resolutions of thanks had been passed, and the President had made a short farewell address, the Association adjourned.—*Boston Medical and Surgical Journal.*

